# Effects on cognitive function and mood state due to interventions involving finger movements in healthy older adults

**DOI:** 10.1093/geroni/igaf122.2446

**Published:** 2025-12-31

**Authors:** Atsuko Hayashi, Momoka Ijima, Keitaro Ito, Sinan Chen, Masahide Nakamura

**Affiliations:** Kobe University, Kobe, Hyogo, Japan; Kobe University, Kobe, Hyogo, Japan; Kobe University, Kobe, Hyogo, Japan; Kobe University, Kobe, Hyogo, Japan; Kobe University, Kobe, Hyogo, Japan

## Abstract

Previous studies have reported relationships between hand dexterity and cognitive functions. However, there are few studies examining effects of finger exercise interventions on cognitive functions. Here we administered working memory(WM) tasks involving finger movements as interventions in healthy older subjects and investigated changes in cognitive functions and mood state due to the interventions. The participants were 71 healthy older people in total (a cross-over study). They were randomly assigned to the following three intervention groups; Group A performed a WM task using numbers and Japanese kana characters accompanied by finger movements; Group B performed finger movements in accordance with the presented numbers or kana; Group C were allocated to the WM task without finger movements. Cognitive functions [Trail Making Test(TMT)-A/B, Digit/Tapping Span] and mood states (the Two-Dimension Mood Scale) were evaluated. In the comparison of before and after the intervention, group A significantly improved the time required for TMT-A/B. Group B showed a significant decrease in the time required for TMT-B. In group C, the score of Digit Span was increased and all groups showed increased arousal level and decreased mood stability significantly. In between-group comparisons, no significant changes were observed in all evaluations. These results suggest that the interventions involving finger movements may improve cognitive functions such as attention and executive functions and working memory in healthy older people. The intervention tasks might affect in mood states regardless of finger exercises. In conclusion, it is suggested that finger exercises may be used as intervention tools to improve cognitive functions.

